# Simultaneous Ipsilateral Quadriceps and Triceps Tendon Rupture in a Patient with End-Stage Renal Failure

**DOI:** 10.1155/2018/7602096

**Published:** 2018-08-05

**Authors:** Kevin Moerenhout, Georgios Gkagkalis, Benoit Benoit, Georges Yves Laflamme

**Affiliations:** ^1^Orthopedic Surgery, Department of Surgery, 5400 Boul Gouin O, Hôpital du Sacré-Cœur de Montréal, Montréal, QC, Canada H4J 1C5; ^2^Service of Orthopaedics and Traumatology, Lausanne University Hospital, Rue du Bugnon 46, CH-1011 Lausanne, Switzerland

## Abstract

**Introduction:**

Quadriceps tendon ruptures (QTR) frequently occur in patients with end-stage renal failure, while triceps brachii tendon ruptures (TTR) are less common. This is the first properly documented report of a simultaneous ipsilateral traumatic rupture of both of these tendons.

**Case Report:**

A 50-year-old patient, on hemodialysis for end-stage renal failure, fell on his right side. He presented with sudden right knee and elbow pain, with functional impairment of both joints. X-rays showed avulsion-like osseous lesions on the olecranon and patella with a low-riding patella. Ultrasound confirmed complete quadriceps and triceps avulsion ruptures. Both lesions were treated surgically. Fixation was performed with anchors using the Krackow suture technique for both tendons. Postoperative clinical and radiological results were satisfactory, and follow-up was uneventful. The patient regained his preinjury functional level with a complete range of motion of both his knee and elbow.

**Discussion:**

Isolated QTR and TTR are frequent lesions in chronic renal failure patients treated with hemodialysis. Simultaneous ipsilateral rupture of both tendons however is extremely rare and should therefore not be overlooked. Surgical treatment is recommended for complete ruptures.

## 1. Introduction

Tendon ruptures are known to be associated with end-stage renal failure and are frequently found in patients treated with hemodialysis. The most frequently involved tendons are the quadriceps tendon, the Achilles tendon, the patellar tendon, and the triceps tendon. Usually, a tendon rupture is isolated, although some studies report simultaneous bilateral quadriceps [1] or triceps tendon ruptures [2–4]. Sequential quadriceps and triceps tendons [5], even bilateral cases [6], have also been described. However, we have found no other report, except for one case of spontaneous rupture due to a hypocalcemia-induced tetany [7], of a simultaneous ipsilateral quadriceps (QTR) and triceps tendon rupture (TTR) following trauma. Our report presents a case of simultaneous ipsilateral quadriceps and triceps tendon rupture with clinical findings, treatment options, pre- and postoperative imagery, rehabilitation program, and functional outcome.

## 2. Case Presentation

**Figure 1:**
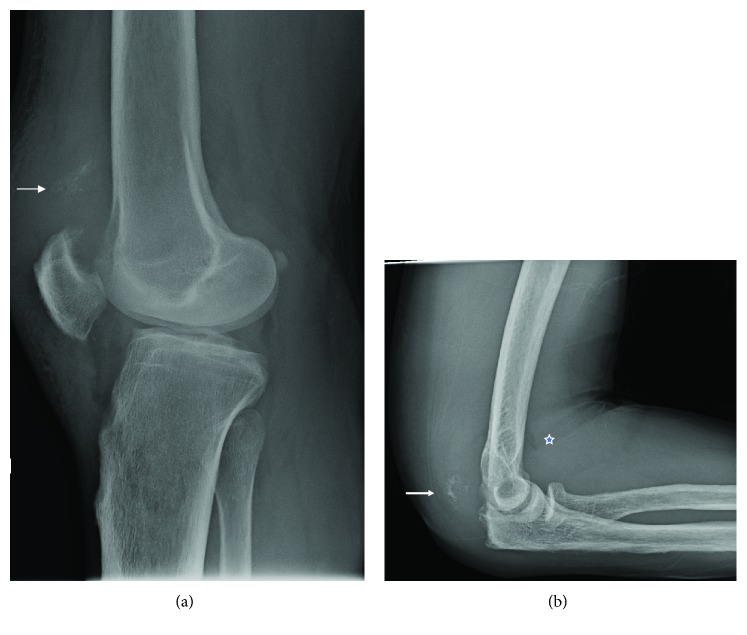
(a) Lateral X-rays of the right knee showing osseous flake (arrow) proximal to the low-riding patella, anterior tilt of proximal patella, and veiled aspect of the patellar tendon. (b) Lateral X-rays of the right elbow showing osseous flake (arrow) of the proximal olecranon and intra-articular effusion with typical sail sign (star).

**Figure 2:**
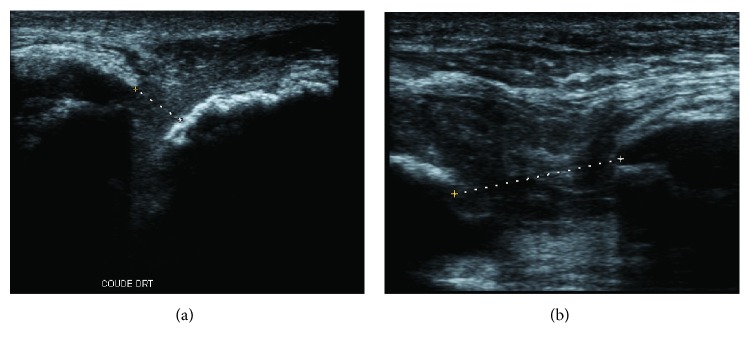
Ultrasound showing the tendinous gap of the triceps, extending further upon joint flexion: (a) gap in extension and (b) gap in flexion.

We report the case of a 50-year-old patient who sustained a fall after slipping on ice. The patient was in the hemodialysis program for end-stage renal failure due to malignant hypertension-induced microangiopathy. He presented with sudden pain to his right knee and elbow, with functional impairment of both joints. X-rays showed avulsion-like osseous lesions on the olecranon and patella with a low-riding patella (Figure 1). Ultrasound confirmed complete quadriceps and triceps rupture avulsions (Figure 2).

Laboratory findings upon admission to the hospital were as follows: GFR: <15 ml/min, blood urea: 24.1 mg/ml (normal range: 7–20), creatinine: 897 *μ*mol/l (normal range: 60–110), sodium: 138 mg/ml, potassium: 5.1 mg/ml (normal range: 3.5–5.0), serum calcium: 2.4 mg/ml, chloride: 98 mg/ml, magnesium: 1.06 mg/ml, phosphorus: 1.15 mg/ml, total protein: 61 g/l, albumin: 33 g/l (normal range: 35–55), and parathormone: 349 pmol/l (normal range: 1.2–5.8). The medical treatment of the patient consisted of darbepoetin alfa (800 mcg/week), sevelamer (800 mg 3x/day), alphacalcidol (0.25 mcg/day), perindopril (8 mg/day), amlodipine (10 mg/day), bisoprolol (5 mg/day), aspirin (80 mg/day), and calcium supplements (500 mg/day).

**Figure 3:**
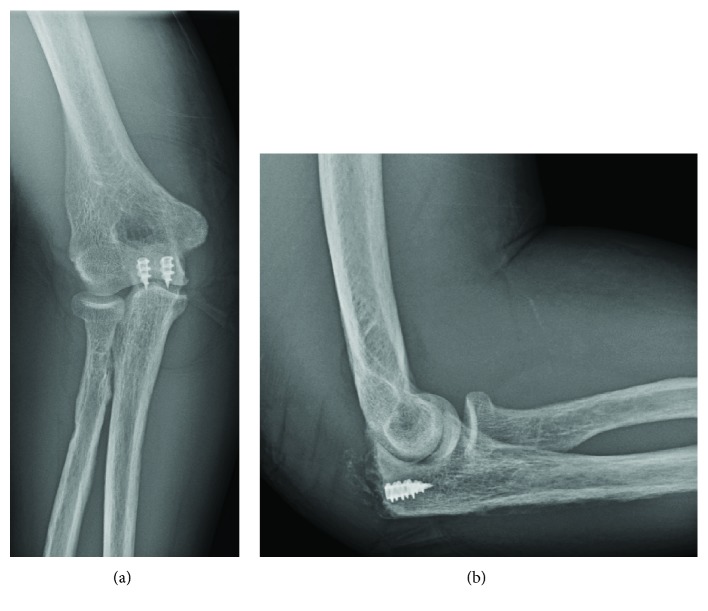
Postoperative (a) AP and (b) lateral X-ray of the elbow joint, showing 2 anchors in the olecranon, where sutures are anchored.

**Figure 4:**
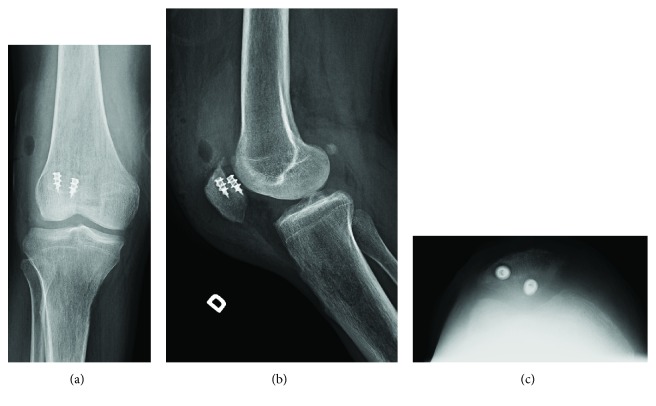
Postoperative (a) AP, (b) lateral, and (c) sunshine view of X-rays of the knee joint, showing 2 anchors in the proximal patella.

The general medical condition was adequate, allowing surgical treatment. The patient was placed in the supine position, and both joints were draped during the same session. Both triceps and quadriceps presented an avulsion-like rupture at their osteotendinous junction, with an otherwise normal appearance macroscopically. Two anchors were inserted in the olecranon and another two in the proximal patella. Then, the Krackow suture technique was used on both tendons with nonabsorbable sutures. Nonviable tendon extremities were debrided. Postoperative X-rays were satisfactory (Figures 3 and 4).

The rehabilitation protocol consisted of unrestricted elbow mobility without weight bearing for 6 weeks and flexion being limited to 90 degrees during this period. Knee rehabilitation consisted of total weight bearing with an extension brace and progressive flexion during the first 6 weeks following surgery (10 days at 0°, 30°, 60°, and 90°). There were no restrictions after the 6-week clinical control. AOFAS and OKS at 3 months were similar to the preoperative evaluation. The patient regained a full range of motion of the elbow and knee as well as preinjury activity level.

## 3. Discussion

Steiner and Palmer described the first case of bilateral QTR in the context of renal failure more than seven decades ago [8]. Finlayson et al. showed a correlation between connective tissue elastosis and chronic acidosis in 1964 [9]. Since then, several case reports of single tendon ruptures have been published. Bilateral QTR or TTR ruptures have also been reported, and there was even a case of bilateral Achilles tendon rupture followed by bilateral TTR, due to oversolicitation during rehabilitation, in a hemodialysis patient [6]. There are also reports of cases of combined TTR, mainly caused by a fall on the outstretched hands, creating excessive triceps contracture. A rare case of multiple tendon ruptures was published in 1965 by Murphy and McPhee [10], describing a patient with combined quadriceps, patellar, and triceps brachii tendon ruptures. Various rupture combinations of the tendons around the knee, patella, or quadriceps have also been largely reported. They are due to a massive contraction of the knee extensors when forced flexion is applied or when falling on the knee. Mont et al. published a case of hypocalcemic tetany resulting in bilateral QTR and unilateral TTR, but this is considered a spontaneous and not a traumatic tendon rupture [7]. A combination of QTR and TTR was described by Lotem et al., but they did not adequately document whether the lesions were complete or incomplete and whether they were simultaneous or sequential [11]. Despite the fact that many cases of single, bilateral, or even sequential tendon ruptures have already been described in the literature, we consider our case a novel presentation of traumatic quadriceps and triceps tendon rupture associated with end-stage renal disease.

The etiology of tendon rupture in the context of chronic renal failure requiring hemodialysis includes secondary hyperparathyroidism, corticosteroid treatment, metabolic acidosis, fluoroquinolone administration, and postdialysis amyloidosis [12–15]. The present case showed secondary hyperparathyroidism, which is a typical complication of chronic kidney disease. Indeed, the decreased glomerular filtration rate and calcium level combined with a high phosphorus blood level lead to increased parathyroid hormone (PTH) levels in chronic kidney disease patients. This high level of PTH stimulates osteoclast activity and could explain the traumatic avulsion of the tendinous insertion in this patient.

Adequate clinical evaluation is essential in order to recognize a quadriceps or triceps tendon rupture. Perfitt et al. published that 67% of patients with quadriceps ruptures were misdiagnosed at their first presentation, which can lead to inferior clinical results [16]. Diagnosis is mostly clinical, with the patient presenting with knee pain and a palpable gap (usually) and being unable to perform an extension. Full extension may nevertheless be possible if medial and lateral retinacula are intact. X-rays should be taken to rule out a fracture, while tendon rupture can be confirmed by ultrasound or MR imaging. Ultrasound has the advantage of being a low-cost exam that is readily available virtually everywhere. However, the results are highly dependent on the level of experience of the radiologist and while being highly sensitive, it has a low specificity. MRI, on the other hand, has a high sensitivity/specificity and a high positive predictive value in detecting quadriceps or triceps tendon rupture. Unfortunately, it is more expensive, less promptly accessible, and not yet universally available [16, 17].

Complete tendon ruptures must be treated surgically in order to regain complete function recovery [12, 18–20]. The two most common techniques are transosseous sutures (gold standard) or suture anchors, which have both yielded excellent outcomes [19, 21–23]. We opted for suture anchors as we have achieved fast and reproducible results using this method.

There is no consensus on the postoperative rehabilitation protocol when it comes to the range of motion and interval [24, 25]. We opted for a more aggressive protocol with progressive reeducation of the knee and a gradual increase in mobility. Full-weight bearing was permitted immediately after surgery. In terms of elbow mobility, 90° of flexion, without lifting loads over 5 kg, was permitted. This rehabilitation program was successful, and the patient regained a complete range of motion and strength.

In conclusion, isolated quadriceps and triceps tendon ruptures are frequently reported in chronic renal failure patients treated with hemodialysis. Such patients should be informed on the risk of tendinopathy associated with their renal disease, and surgeon awareness should be high in cases of tendon ruptures in this particular group. Diagnosis is made clinically and can be confirmed by ultrasound or MRI. This is the first report of a simultaneous ipsilateral rupture of both quadriceps and triceps tendons following trauma. Treatment should be surgical if the patient's general medical condition allows it.
